# Human tissue models for a human disease: what are the barriers?

**DOI:** 10.1136/thoraxjnl-2014-206648

**Published:** 2015-01-28

**Authors:** Joanna Edwards, Maria Belvisi, Sven-Erik Dahlen, Stephen Holgate, Anthony Holmes

**Affiliations:** 1 National Centre for the Replacement, Refinement and Reduction of Animals in Research, London, UK; 2 Faculty of Medicine, National Heart & Lung Institute, Imperial College London, London, UK; 3 Institute of Environmental Medicine, Karolinska Institute, Stockholm, Sweden; 4 Faculty of Medicine, University of Southampton, Southampton, UK

**Keywords:** Asthma, Asthma Mechanisms, Asthma Pharmacology

## Abstract

Asthma represents an area of significant unmet medical need, with few new drugs making it to the clinic in the past 50 years. Much asthma research is currently carried out in non-human models. However, as asthma is a uniquely human condition, it is difficult to translate findings from these models to efficacious therapies. Based on the results of a survey of the UK asthma research community carried out jointly between the NC3Rs, Asthma UK, the UK Respiratory Research Collaborative and the Human Tissue Authority, we propose that more emphasis be placed on the use of human tissue studies to provide more relevant models that better translate to the clinic and which reduce the reliance of the asthma community on less predictive animal models.

## Introduction

Inhaled corticosteroids and β-2 agonists have been the first-line treatment for asthma since the 1960s, despite almost half a century of targeted research.^1^ These control the symptoms of the majority of patients, who experience mild-to-moderate forms of the disease. However, 5%–10% of the asthma population exhibit severe symptoms that are not adequately controlled by conventional inhaled therapy.^2^ While this represents a small proportion of asthma sufferers, they account for almost half of healthcare costs related to the disease and the majority of asthma-related deaths.^3^ There is a great unmet need to discover new treatments in severe asthma and also for the different sub-phenotypes that are progressively recognised to represent distinct disease mechanisms.^4^

Many promising drugs that perform well in preclinical animal studies fail in humans due to lack of safety and/or efficacy, suggesting the current preclinical testing strategy, focusing largely on murine in vivo models, which do not recapitulate the complexity of the human disease, is not sophisticated enough to meet today's respiratory drug development needs.^5^ A new approach to drug discovery and development in this area is necessary.

Although some facets of the asthmatic phenotype can be modelled in certain animal species, no animals that are commonly used to study the condition (including mice, rats, guinea pigs and rabbits) develop an asthma-like syndrome which completely correlates to the human disease. As asthma is a disease unique to humans, the development and application of human tissue-based approaches with which to study the disease should be considered a priority.

## Methods

The NC3Rs, working with Asthma UK, the UK Respiratory Research Collaborative and the Human Tissue Authority, recently surveyed the UK asthma research community to better understand the extent of human tissue use in this area.

The survey was distributed as an online questionnaire through the partner networks and others, including the British Thoracic Society, the British Lung Foundation and the British Association for Lung Research. The survey was completed by 59 individuals from academia, pharmaceutical companies, small and medium enterprises and the National Health Service.

The survey was divided into sections to capture information on:

1. The way human lung tissue is currently used in asthma research;
2. The level of knowledge surrounding the regulatory requirements and guidance on human tissue use;
3. The perceived barriers to wider uptake of human tissue-based approaches in asthma research.

## Results

Only 14% of people surveyed do not currently use human tissue in their asthma research programmes. Of those who do (86%), 16% use it exclusively, while the majority use it in conjunction with immortalised cells or cryopreserved material (47%) or alongside animal studies (37%).

The type of human tissue used is predominantly limited to that which is easy to access, with 86% of respondents using either normal or diseased primary cells, 47% using sputum and 53% using biofluids. In contrast, the percentage using whole lung is much lower at 24%.

The type of tissue currently used in asthma research contrasts strongly with the type of tissue that researchers would like to use if they could access it, with only 13% wanting to use primary cells, while 44% expressed a desire to use whole lungs. The reasons given for not being able to use these types of tissue focused on the difficulty in accessing them, with normal tissue being in such high demand that there is not enough available to support studies. Respondents also indicated a desire to use larger tissue samples, such as wedge resections or whole lobes for functional experiments on isolated airways, blood vessels and other tissue components.

**Figure 1:**
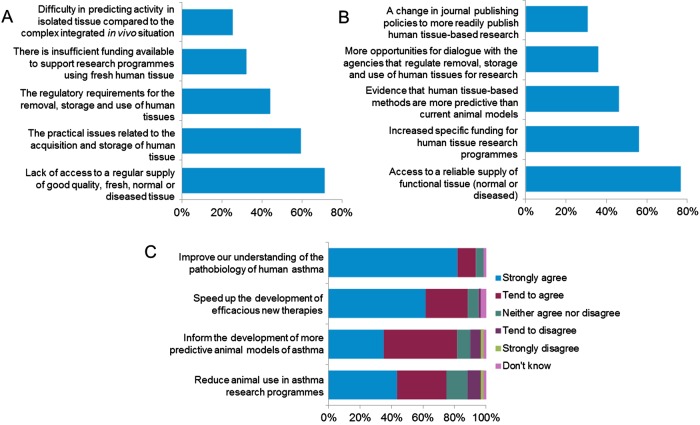
(A) The top five barriers to the use of human tissue in asthma research. (B) Top five changes that would enable researchers to use more human tissue in their asthma research programmes. (C) Extent to which respondents agree or disagree that better access to, and wider use of, fresh human tissue would (i) reduce the number of animals used for asthma research, (ii) inform the development of more predictive animal models, (iii) speed up the development of efficacious new therapies and (iv) improve our understanding of the pathobiology of asthma.

The survey highlighted a number of barriers to using fresh human tissue in asthma research (figure 1A). The majority of those questioned (71%) were concerned about the availability of either normal or diseased fresh human tissue. Another widely considered barrier is the practical issues related to the acquisition and storage of tissue (59%). Respondents considered that increasing access to a reliable supply of normal and diseased human tissue (71%), increased specific funding for human tissue research programmes (56%) and evidence that human tissue-based methods are more predictive than current animal models (46%) would enable greater adoption of human tissue methods as part of asthma research programmes (figure 1B). Other opportunities for increasing adoption of human tissue approaches are considered subsequently.

The impacts of wider adoption of human tissue for asthma research were far reaching, with respondents agreeing that this would reduce animal use, result in more predictive models, lead to new therapies and improve our understanding of the human disease (figure 1C).

## Discussion

The survey suggests that there is already widespread use of human tissue in asthma research, with over 86% of respondents reporting that they use human tissue in some capacity. The survey also highlights that human tissue is used in a wide variety of research areas, including immunology, physiology, pharmacology, genetics and epigenetics, and compound evaluation. However, it is clear that most of this human tissue use is limited to isolation of individual cells rather than studies of airways or other components of the lung allowing for experiments in complex intact tissue.

The consensus among the researchers questioned is that there are clear benefits that can be realised by increasing the use of human tissue in asthma research. These include a greater understanding of the pathobiology of asthma, quicker development of efficacious new therapies to treat the disease, the development of more predictive animal models of asthma and a reduction in the overall number of animals used in asthma research programmes.

So what is preventing more human tissue use? The most common barrier is access, particularly to normal human tissue and also to diseased tissue. The major obstacle to an effective supply of human tissues is the logistical hurdles that need to be overcome. A lot of human lung tissue is being disposed of in hospitals all over the country. There is a need for greater cooperation between pathologists, transplant surgeons and researchers, and special research funding for resources to manage the collection, processing and transport of tissues for research without compromising the requirements of the caregivers. Owing to these hurdles, 28% of those using human tissue use a commercial supplier, either in the UK or internationally, to ensure a regular supply of lung tissue. This suggests there is a need for a more widely accessible UK-based lung tissue bio-resource or tissue network to obtain fresh human tissue, which 83% of respondents indicate they would be likely to access.

The regulatory requirements for the removal, storage and use of human tissues are seen as a barrier to increased human tissue use by 44% of respondents. However, the survey results suggest that this is at least partly a perceived barrier, as there was a lack of knowledge surrounding some aspects of the regulations. The licensing requirements concerning the storage and use of human tissue for research, and the requirements for consent to use and store human tissue for research purposes were considered particularly unclear. As many as 42% of respondents felt there was an insufficient level of information available on the regulatory framework surrounding the collection and use of human tissue. A large percentage (68%) of respondents felt that the regulatory requirements hold up research unnecessarily, with a similar proportion reporting that requirements are complicated and difficult to understand (70%), and that they are not streamlined (75%). This information is easily accessible online and represents one area where better dissemination and improved awareness of existing information could potentially have a significant impact on the uptake of human-tissue use in asthma research.

Journals also have a role to play in enabling more widespread human tissue use, according to 51% of survey respondents. A greater willingness on the part of journals to publish human tissue research without accompanying animal model data would increase the evidence base to support the use of these models and provide confidence to encourage their wider uptake.

The current survey illustrates the views of a representative proportion of the asthma research community. It provides important evidence that a more concerted effort is needed to support asthma researchers in adopting human tissue-based approaches. Many barriers exist to this, and some of these may be more easily overcome than others, but the potential benefits to the science base, drug development and the 3Rs (the replacement, reduction and refinement of animals in research) are many. The asthma research community needs to come together to share information on how these barriers can be overcome, and it is important that funders, journal editors and regulators all play their part in supporting this. Additionally, there needs to be a meeting of minds between caregivers and researchers to ensure that as much as possible of the human tissue being collected in hospitals, which is surplus to healthcare needs, is made available to the research community. Recognising this need, the NC3Rs is developing tools to help researchers access relevant information to expedite the wider adoption of human tissue-based models in basic research and drug development. In conclusion, we believe it is important that the major funding bodies and other key stakeholders give higher priority to allocate the resources required to enhance and facilitate respiratory research using human tissues.

## References

[1] Crompton G A brief history of inhaled asthma therapy over the last fifty years. Prim Care Respir J 2006;15:326–31. 10.1016/j.pcrj.2006.09.002 17092772 PMC6730840

[2] Sullivan SD, Rasouliyan L, Russo PA, et al Extent, patterns, and burden of uncontrolled disease in severe or difficult-to-treat asthma. Allergy 2007;62:126–33. 17298420 10.1111/j.1398-9995.2006.01254.x

[3] Smith K, Warholak T, Armstrong E, et al Evaluation of risk factors and health outcomes among persons with asthma. J Asthma 2009;46:234–7. 10.1080/02770900802627294 19373629

[4] Wenzel SE Asthma phenotypes: the evolution from clinical to molecular approaches. Nat Med 2012;18:716–25. 10.1038/nm.2678 22561835

[5] Holmes AM, Solari R, Holgate ST Animal models of asthma: value, limitations and opportunities for alternative approaches. Drug Discov Today 2011;16:659–70. 10.1016/j.drudis.2011.05.014 21723955

